# The Influence of Sex Steroid Hormones in the Immunopathology of Experimental Pulmonary Tuberculosis

**DOI:** 10.1371/journal.pone.0093831

**Published:** 2014-04-10

**Authors:** Estela Isabel Bini, Dulce Mata Espinosa, Brenda Marquina Castillo, Jorge Barrios Payán, Darío Colucci, Alejandro Francisco Cruz, Zyanya Lucía Zatarain, Edgar Alfonseca, Marta Romano Pardo, Oscar Bottasso, Rogelio Hernández Pando

**Affiliations:** 1 Experimental Pathology Section. Department of Pathology, National Institute of Medical Sciences and Nutrition “Salvador Zubirán”, México City, México; 2 Immunology Institute, Medical Sciences Faculty, Santa Fe, Rosario, Argentina; 3 Physiology and Biophysic Department. CINVESTAV, Mexico City, Mexico; National Institutes of Health, United States of America

## Abstract

The relation between men and women suffering pulmonary tuberculosis is 7/3 in favor to males. Sex hormones could be a significant factor for this difference, considering that testosterone impairs macrophage activation and pro-inflammatory cytokines production, while estrogens are proinflammatory mediator’s inducer. The aim of this work was to compare the evolution of tuberculosis in male and female mice using a model of progressive disease. BALB/c mice, male and female were randomized into two groups: castrated or sham-operated, and infected by the intratracheal route with a high dose of *Mycobacterium tuberculosis* strain H37Rv. Mice were euthanized at different time points and in their lungs were determined bacilli loads, inflammation, cytokines expression, survival and testosterone levels in serum. Non-castrated male mice showed significant higher mortality and bacilli burdens during late disease than female and castrated male animals. Compared to males, females and castrated males exhibited significant higher inflammation in all lung compartments, earlier formation of granulomas and pneumonia, while between castrated and non-castrated females there were not significant differences. Females and castrated males expressed significant higher TNF-α, IFN γ, IL12, iNOS and IL17 than non-castrated males during the first month of infection. Serum Testosterone of males showed higher concentration during late infection. Orchidectomy at day 60 post-infection produced a significant decrease of bacilli burdens in coexistence with higher expression of TNFα, IL-12 and IFNγ. Thus, male mice are more susceptible to tuberculosis than females and this was prevented by castration suggesting that testosterone could be a tuberculosis susceptibility factor.

## Introduction

Males of many species are more susceptible than females to infections caused by certain parasites, fungi, bacteria, and viruses [1]. In the particular case of tuberculosis (TB) and in almost all but especially in developing countries, TB is twice as common in men than in women (male/female ratio of 1.9+/−0.6 for the world case notification) [2]. This difference has been attributed to biological and epidemiological characteristics [3], [4], aswell as socioeconomic and cultural barriers in the access to health care [5]. It is interesting to note that this TB gender difference is seen in adults of all ages, but not in children or young adolescents [6]. This observation suggests the participation of biological factors, particularly the well known regulatory activities that the steroid sex hormones have on the immune cells. Macrophages and lymphocytes have receptors for androgens, estrogens and progesterone [7]. These hormones participate in macrophages and lymphocytes development and function, as well as in the outcome of diverse diseases, including infectious diseases [8]. Females have higher antibodies levels in sera [9] and exhibited stronger immune responses after immunization than males [10], [11]. Moreover, women of all ages show significantly lower rates of infection and resultant mortality than men. This difference has been associated to important differences in the inflammatory response and is apparently advantageous against infection, but unfavorable in the immune response against self structures provoking in females a higher rate of autoimmune diseases [12], [13].

Testosterone, the main circulating androgen in men and progesterone a hormone associated with the maintenance of pregnancy, are immunosuppressive. Both hormones impair macrophage activation [14] and could play a detrimental role in TB [15]. In contrast, in physiological concentration estrogens are considered pro-inflammatory mediators that stimulate the production of TNF-α [16], and interact with the IFN-γ promoter [17].

The ability of estrogens to drive pro-inflammatory Th-1 associated immune responses and that of testosterone to inhibit them may help to explain why females have a lower incidence of infectious diseases such as TB [6], but surprisingly this subject has not been fully studied in TB experimental models. Early reports for saprophytic mycobacterial infections showed that female mice are more resistant to infection with *M. intracellulare* and *M. marinum* [18], [19]. The treatment of females or castrated males with testosterone increases their susceptibility to *M. marinum*, and estradiol treatment abolishes the higher susceptibility of ovariectomized mice to *M. avium* [20], but as far as we know there are no reports of similar experiments with *M. tuberculosis.*

This work aimed at comparing the course of infection between males and females, castrated and non-castrated animals using a model of progressive pulmonary TB in BALB/c mice infected by intratracheal route with a high dose of the laboratory *M. tuberculosis* strain H37Rv. We assessed survival, the pulmonary inflammatory response, bacillary loads, immune response (analizing cytokine gene expression determined by RT-PCR) and serum testosterone levels. In order to investigate the effects of the testosterone supression during late progressive disease, we also analized the same parameters in the lungs of males BALB/c mice castrated two months after infection and euthanized one month later.

## Materials and Methods

### Ethics Statements

All the animal work was done according to the guidelines of the Mexican constitution law NOM 062-200-1999, and approval of the Ethical Committee for Experimentation in Animals of the National Institute of Medical Sciences and Nutrition in Mexico (CINVA), permit number: 224. All surgery was performed under sevofluorane anaesthesia, and all efforts were made to minimize suffering.

### Experimental Model of Progressive Pulmonary TB in BALB/c Mice

The experimental model of progressive pulmonary TB has been described in detail elsewhere [21], [22]. Briefly, the laboratory *Mycobacterium tuberculosis* strain H37Rv (ATCC No. 25618) was grown in Middlebrook 7H9 broth (DIFCO) supplemented with 0.2% (v/v) glycerol, 10% OADC enrichment (DIFCO), and 0.02% (v/v) Tween-80 at 37°C. Mid log-phase cultures were used for all experiments. Mycobacteria were counted and stored at –80°C until use. Bacterial aliquots were thawed and pulse-sonicated to remove clumps.

A group of 45 BALB/c male mice, 8-week-old and 21–23 gr of weight, were gonadectomized by removing both testes trough a surgical incision along the median line of the scrotum under anesthestesia in gas chamber using 0.2 ml per mouse of sevofluorane. Other group with the same number of animals corresponded to the sham group; in these mice, a midline incision along the scrotum was made and through it both testes were pulled out and then reinserted. The skin was stiched with sterile silk.

In a similar way, a group of 45 BALB/c female mice, 8-week-old and 21–23 gr of weight, was gonadectomized by removing both ovaries through bilateral incisions over the dorsum under anesthestesia in gas chamber using 0.2 ml per mouse of sevofluorane. In the sham operation group, ovaries were identified and the surgical incision was then stitched with sterile silk. After two weeks for recovering, all the animals were anesthetized in gas chamber using 0.1 ml per mouse of sevofluorane, and infected through endo-tracheal instillation with 2.5×10^5^ live bacilli into a cabinet level III of biosecurity. Mice were maintained in vertical position until spontaneous recovery. Infected mice were maintained in groups of five in cages fitted with micro-isolators connected to negative pressure in biosafety level III facilities. Animals were kept with lights on from 6.00–18.00 hr and feed with sterilized chow ad libitum and drinking autoclaved water. Groups of five animals were euthanized into a cabinet biosecurity level III at 1, 3, 7, 14, 21, 28, and 60 days post infection by exsanguinations under anaesthesia with 56 mg/Kg of intraperitoneal pentobarbital. Three lungs, right or left, per time point were fixed and prepared for histopathological studies. After eliminating hilar lymph nodes and thymic tissues, seven lungs more were frozen and kept to −70°C for bacilli loads determination and gene expression studies in two separated experiments. Ten animals per group were left untouched and the mortality was recorded in order to construct survival graphs. Animals were monitored every day and when they showed abnormalities such as respiratory insuficiency, accentuated caquexia or total immobilization they were humanely euthanized under anesthesia induced by intraperitoneal pentobarbital.

### Preparation of Lung Tissue for Histological Analysis and Morphometry

Lungs from infected mice were perfused with 10% formaldehyde diluted with in PBS via the trachea, fixed for 24 hr and embedded in paraffin. Sections, 5 μm thick, taken through the hilus were mounted on glass slides, deparaffinized, and stained with hematoxylin and eosin. For quantification of inflammatory infiltrates, at least three different mice lungs per time point in two different experiments were evaluated. Ten random microscopy fields were selected at ×20 magnification. The area occupied by the inflammatory infiltrate around the venules (100 μ of diameter), bronchi (150–200 μ of diameter) and in the alveolar-capillary interstitium, as well as the granuloma size and the lung surface occupied by pneumonia were measured in a Q-win Leica 500 morphometry equipment [23].

### Determination of Colony-Forming Units (CFU) in Infected Lungs

Right or left lungs from four mice at each time point, in two separate experiments, were used for colony counting. Lungs were homogenized with a Polytron (Kinematica, Luzern, Switzerland) in sterile 50 ml tubes containing 3 ml of isotonic saline. Four dilutions of each homogenate were spread onto duplicate plates containing Bacto Middlebrook 7H10 agar (Difco Labs, Detroit MI, USA) enriched with oleic acid, albumin, catalase and dextrose. Incubation time and colony counting was 21 days [23].

### Real Time PCR Analysis of Cytokines in Lung Homogenates

Left or right lung lobes from three different mice per group in two different experiments were used to isolate mRNA using the RNeasy Mini Kit (Qiagen), according to recommendations of the manufacturer. Quality and quantity of RNA were evaluated through spectrophotometry (260/280) and on agarose gels. Reverse transcription of the mRNA was performed using 5 μg RNA, oligo-dT, and the Omniscript kit (Qiagen, Inc). Real-time PCR was performed using the 7500 real time PCR system (Applied Biosystems, USA) and Quantitect SYBR Green Mastermix kit (Qiagen). Standard curves of quantified and diluted PCR product, as well as negative controls, were included in each PCR run. Specific primers for genes encoding acidic ribosomal protein (RLP0) as house keeping gene (FWD: 5′-CTC TCG CTT TCT GGA GGG TG-3′; RV: 5′-ACG CGC TTG TAC CCA TTG AT-3′), TNF-α, IFN-γ, IL-12, iNOS, IL17, were designed using the program Primer Express (Applied Biosystems, USA) [24].

Cycling conditions used were: initial denaturation at 95°C for 15 min, followed by 40 cycles at 95°C for 20 sec, 60°C for 20 sec, 72°C for 34 sec. Quantities of the specific mRNA in the sample were measured according to the corresponding gene specific standard. The mRNA copy number of each cytokine was related to one million copies of mRNA encoding the RLP0 gene [25].

### Kinetics of Testosterone Concentration in Serum and the Effect of Gonadectomized Male Mice during Late Progressive Disease

Serum testosterone levels from male mice were measured using an especific testosterone ELISA kit for mouse (EIA 1559, DRG Instruments, GmbH, Germany), following the recommendations of the manufacturer and reading at 450 nm in a Tecan Sunrise microtiter plate reader. Two independent experiment of gonadectomy during late progressive disease were carried out in 20 males 8-week-old BALB/c mice infected via the intratracheal route as above described. Two monts after infection, 10 animals were castrated and other group with the same number of animals was sham operated as described above. On day 30 after castration (90 day postinfection), animals were euthanized and pulmonary morphometry, bacilli loads and cytokines expression were determined as described above.

### Statistical Analysis

Data are presented as the mean ± standard deviation. Differences among groups were evaluated by the Anova F test, whereas the Student *t* test was used for further analysis among-group differences. Survival curves were analyzed with Kaplan Meir plots and the Log Rank test. An associated probability lower than 0.05, was considered significant.

## Results

### Effect of Mice Gender and Gonadectomy on Survival and Bacterial Numbers after Infection with *M. Tuberculosis*

**Figure 1:**
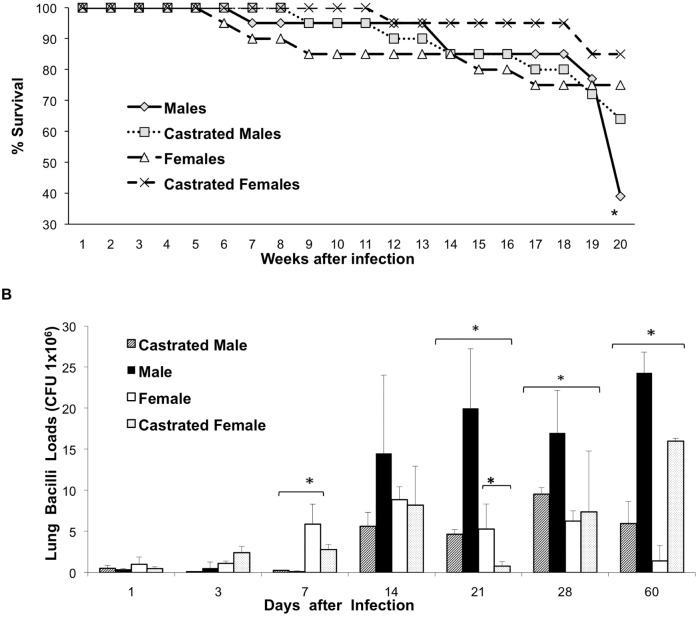
Survival and pulmonary bacilli loads comparisons among male and female BALB/c mice, castrated and non-castrated. Mice were infected by intratracheal route with *Mycobacterium tuberculosis* H37Rv. A) Survival curve constructed with 20 mice, male mice showed significant lower survival difference when compared with female mice (p<0.005, Log rank test). B) Lung bacterial burdens, mice were sacrificed at the indicated days after infection, and lungs (n = 4 per time point) were used for determination of colony forming units. At late disease, male mice showed significant higher bacilli loads than the other groups. Asterisks represent statistical significance.

In order to study the effect of the gender in the course of experimental TB, groups of non-castrated male (M) and female (F) and castrated (CM, CF) BALB/c mice were infected by the intratracheal route with the reference strain H37Rv. Forty percent of M mice survived after four months of infection. In contrast, infected F mice showed 75% survival rate, a similar survival rate was seen in CF mice, while 60% of CM mice were alive after 120 days post-infection (Fig. 1). These survival rates correlated with the live bacilli burdens in lung homogenates. Since day 14, M exhibited higher number of CFU than CM, F and CF groups. Significant differences were observed since day 21, being the CM group that showed the lowest bacilli loads at day 60 (Fig. 1).

### Effect of Gender and Gonadectomy on the Inflammatory Response during Experimental Pulmonary TB

**Figure 2:**
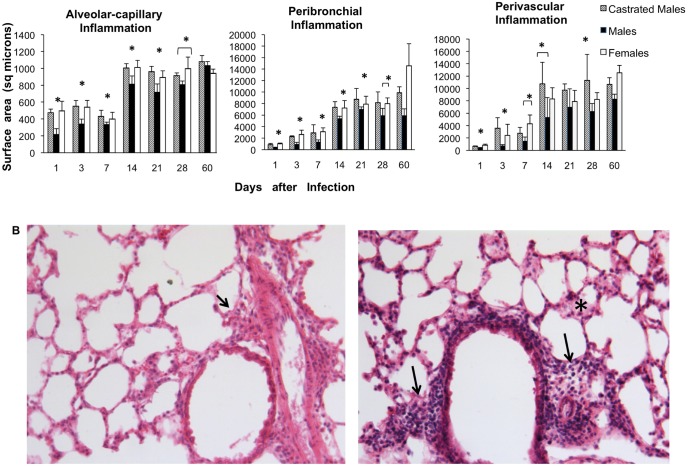
Kinetics of inflammatory infiltrates in the lungs of infected BALB/c mice and representative pulmonary histopathology. A) Kinetics of inflammatory infiltrates in the lungs of noncastrated and castrated male and female mice. Three lungs from the same number of different animals for each time point were prepared for histological analysis; the inflammatory area in each indicated compartment was determined by automated morphometry. Asterisks represent statistical differences. B) Representative histopathology of the lung of mice after 21 days of infection, left figure correspond to a male mouse which shows scarce inflammatory infiltrate in the peri-bronchial and perivascular areas (arrow), while in the right micrograph is a similar histological area from a female mouse with larger inflammatory infiltrates (arrows) including the alveolar-capillary interstitium (asterisk). (hematoxylin/eosin, 100x magnification).

Significantly higher inflammatory infiltrate constituted by lymphocytes and macrophages was seen in all of the lung compartments (alveolar-capillary interstitium, perivascular and peribronchial areas) of CM and F than M, from day 1 up to the 28 included (Fig. 2). F and CF did not show significant differences in the inflammatory response, thus this group is not present in the figure.

**Figure 3:**
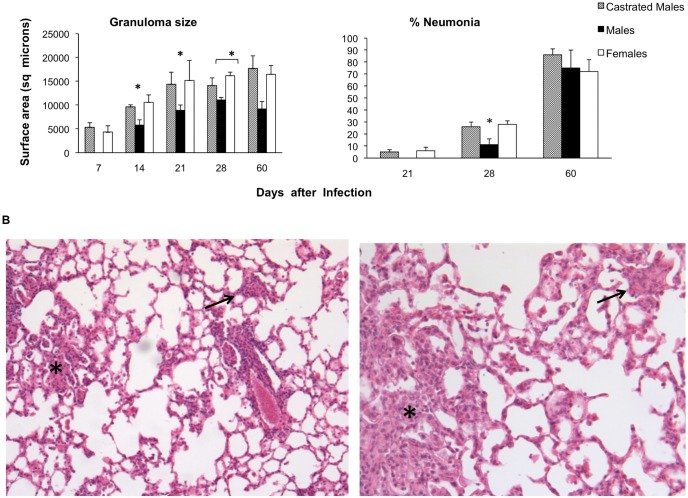
Determination of granuloma size and pneumonia in the lungs after 28 days of infection. A) Size in square microns of granulomas and the percentage of the lung surface affected by pneumonia determined by automated morphometry in at least 3 mice per time point and condition, asterisks represent statistical significance. B) Low power histological fields of the infected lung from a non-castrated male mouse (left figure) and female mouse (right figure) after 28 days of infection. The lung of female mouse shows more extensive area of pneumonia (asterisk) and bigger granulomas (arrows) than the male mouse (hematoxylin/eosin, 10x magnification).

Lungs from CM and F showed well formed, similar size granulomas at one week after infection, while M exhibited smaller granulomas after two weeks of infection and during the rest of the infection (Fig. 3). CM and F mice showed small patches of pneumonia affecting less than 10% of the lung surface after three weeks of infection, while in M group pneumonia started one week later and it was significant lower than in CM and F mice, but during late infection at day 60 lung consolidation was similar among the groups (Fig. 3).

### Cytokines Gene Expression in the Lungs of Infected Mice

**Figure 4:**
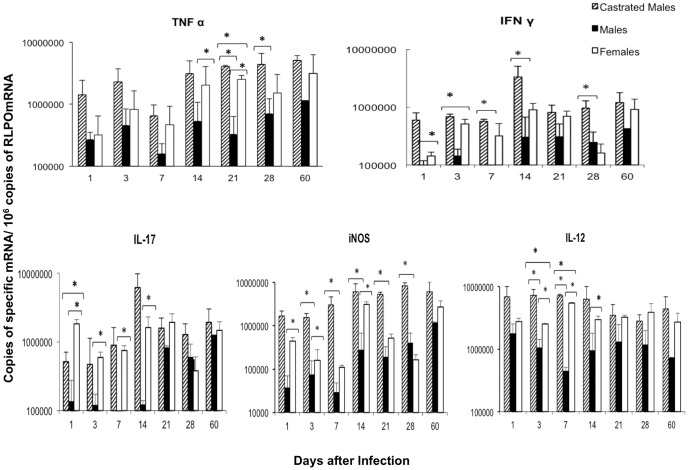
Kinetics of pro-inflammatory cytokines and iNOS gene expression determined by RT-PCR in the infected lungs. Castrated males and noncastrated male and female BALB/c mice were infected with *Mycobacterium tuberculosis* strain H37Rv and euthanized at different time-points. The lungs from three different animals at each time-point were used to determine the gene expression of the indicated cytokine. Asterisks represent statistical significance between the represented groups.

The expression of the pro-inflammatory cytokines TNF-α, IL-12, IFN-γ and IL-17, as well as iNOS was higher in F and CM than in M mice (Fig. 4), while there were not significant differences between F and CF in any of the studied cytokines (data not shown). F and CM showed progressive expression of TNFα, higher than M in all the time points being significant at day 14 and 21, the MC group showed the highest TNFα expression. The expression of IFN-γ, IL-12 and IL-17 was similar in F and CM and higher than in M mice, being significant during early infection, after the first and second weeks: while iNOS was also higher expressed in F and MC than in M, with significant differences during the first month of infection (Fig. 4).

### Testosterone Serum Concentrations and the Effect of Male Castration during Advanced Disease

**Figure 5:**
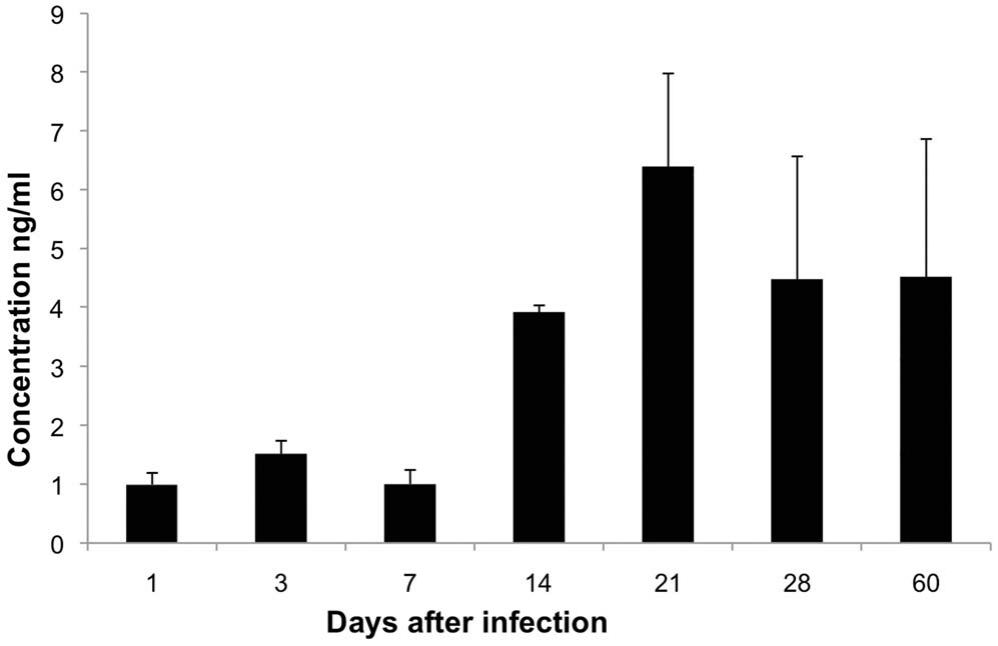
Serum testosterone concentration along pulmonary tuberculosis. Sera from male non-castrated mice were used to determine testosterone by ELISA in the indicated time points after intratracheal infection with *M. tuberculosis* strain H37Rv. Data are expressed as means and standard deviation of six mice per time point.

**Figure 6:**
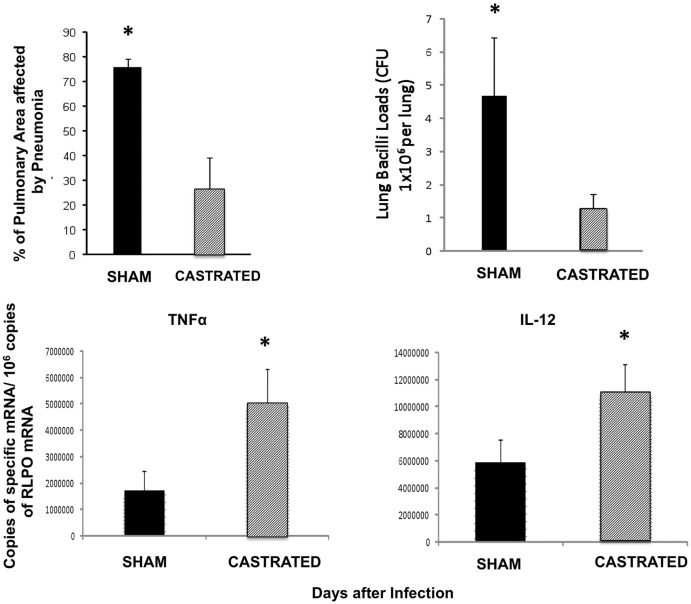
Effect of orchidectomy in BALB/c mice after 60 days of infection. Male mice were infected intratracheally with *M. tuberculosis* strain H37Rv and after two months one group of six animals were castrated and other group was sham operated. One month after castration animals were euthanized and their lungs were used to determine bacilli burdens, tissue damage and gene expression of TNFα and IL-12. Castrated mice showed lower bacilli loads and tissue damage with higher expression of TNFα and IL-12 than control mice. Asterisks represent statistical significance.

The kinetics of serum testosterone concentration along experimental pulmonary TB showed 1–1.5 ng/ml during the first week of infection; at day 14 a four fold increase was determined and it was maintained in similar concentrations until day 60, except at day 21 when the maximal testosterone concentration was detected (6 ng/ml +/−2) (Fig. 5). High testosterone concentrations coincided with progressive pulmonary inflammation due to granulomas and pneumonia formation, suggesting that the increment of testosterone production could contribute to control tissue damage by excessive inflammation but this could also permit bacillary proliferation. In order to investigate this point a group of infected male mice were castrated at day 60 of infection and one month later animals were euthanized to determine pulmonary bacilli burdens, extension of pneumonia and the expression of some pro-inflammatory cytokines. In comparison with the sham control group, gonadectomized male mice showed significant decrease of bacilli loads and pneumonia, as well as higher expression of TNF-α (p = 0,001), IL-12 (p = 0,004) and IFN-γ (non-significant) (Fig. 6).

## Discussion

More than 70% of those individuals who develop active TB are males [6], and wide epidemiological studies in endemic areas from developing countries have shown that males suffer more severe disease, higher rates of recent transmission, more reactivation of latent infection and poorer treatment outcomes [26]. These differences have been attributed to socioeconomic and cultural factors leading to barriers in accessing health care systems, which might cause under notification in women [27]. However, broad epidemiological studies in Mexico [26] and India [28 have demonstrated, that the higher rate of pulmonary TB detected in men is not attributable to unequal access to health services for its diagnostic and treatment, in fact the proportion of women who were screened for TB diagnosis was greater than in men [26]. Although men are more likely to report risk factors that have been associated with exposure to *M. tuberculosis*, such as imprisonment [29], shelter residence [30], alcohol and tobacco consuming [31]. In fact, comprehensive case control studies in West African countries [32] and in Bangladesh [33] concluded, that male gender is a risk factor for TB independent of other examined factors.

The differences in TB rates between females and males have also been attributed to biological factors [6]. In this regard, polymorphisms or mutations in genes located in chromosome X can confer more TB susceptibility in males [34], [35], as well as specific features of metabolism and nutrition related to gender [36], or anatomical and functional differences in the respiratory tract between males and females [37]. Nevertheless, perhaps the most important biological factor associated to different TB susceptibility between males and females is the immune regulatory activities of the sexual hormones [1]. Our results reinforce this statement by the demonstration that male BALB/c mice exhibited higher mortality and bacilli burdens with lower inflammation than female mice and these differences were prevented in castrated male mice.

In general, it seems that androgens have suppressive effects on the celular and humoral immune responses, so they can be considered as natural anti-inflammatory hormones [38], whereas estrogens enhance humoral immunity and affect balance of T and B cells [13]. Regarding to TB this should be important because host control of mycobacterial infection, in both human and mouse, has been associated with Th1 cells and activated macrophages [39]. Experimental studies in mice have demonstrated more male susceptibility to *Mycobacterium lepramurium* [40], *M. avium* complex [20], and *M. marinum* [19]. The treatment with testosterone increases susceptibility to *M. marinum* [19], [18], while administration of estradiol restored the burden of *M. avium* bacilli in CF mice [20]. However, it is important to consider that sex steroids have different functions, even opposite activities, depending on their concentrations. This is particularly evident in females that exhibited significant fluctuations during the menstrual cycle and in specific physiological states such as in pregnancy or menopause. Moreover, high testosterone levels could result in high cortisol levels and an associated reduction in immune function [41].

Our results showed that BALB/c tuberculous M mice died significantly earlier and have higher pulmonary bacilli loads during late disease than tuberculous F mice. Thus, M mice are more susceptible to *M. tuberculosis* infection. Estradiol, the prototype of female steroid hormone has significant influence on inflammation [42], favoring inflammatory cell migration by inducing the expression of mRNA for adhesion molecules (E-selectin, ICAM-1, and VCAM-1) mediated by TNF-α in endothelial cells. This is in agreement with our morphometry results that showed in F mice earlier granuloma formation and higher inflammation in all lung compartments than M mice. F mice also showed alveolar inflammation (pneumonia formation) one week before than M, in coexistence with higher pro-inflammatory cytokines expression and lower bacilli burdens. In late disease, at day 60 post-infection, F mice showed lower bacilli burdens than M but with similar lung consolidation, implying that F could suffer more tissue damage by excessive inflammation. Ovariectomized DBA/2 mice infected by the intratracheal route with *M. avium* showed significantly higher bacilli burdens than sham F mice [20]. In contrast, we did not found differences in terms of mortality and bacilli burdens in tuberculous F and CF mice. These apparent contradictory results could be explained by the participation of diverse factors, such as the different mouse strain and infectious agent with different virulence level (low virulence of *M. avium* and high virulence of *M. tuberculosis)* and antigenic constitution. Indeed, the quality and quantity of antigen stimulation can change the level of sex hormone receptors expressed by macrophages and hence modulating their response [43]. The participation of other sex hormones, such as gonadotrophin releasing hormone which is elevated in gonadectomized animals, can also participate in the protection of CF mice, considering that this hormone promotes both T cell expansion and survival [44]. It is also important to consider the host genetic background, previous studies showed more resistance to atypical mycobacteria infection in C57Bl or DBA/2 M mice than in BALB/c M mice [19].

Interestingly, CM mice showed better survival rate and lower pulmonary bacilli burdens than M mice. Therefore, it seems that the lowered TB resistance in M mice is in part mediated by testosterone. Male castration after puberty in mice increased thymus and spleen size with higher number of peripheral CD4 and CD8 T cells. These cells also showed more vigorous proliferation after specific antigen stimulation and transiently exhibit exaggerated responses to costimulation [38]. These observations were in agreement with our results that showed in tuberculous CM mice, more pulmonary inflammation with higher pro-inflammatory cytokine expression and bigger granulomas than in M mice. CM mice formed granulomas one week before with significant lower bacilli loads than M. Although, lung area affected by pneumonia at late disease was similar in CM than M, suggesting that as in F group, castration could favor excessive inflammation and tissue damage. In this sense, the fact that human males with moderate to severe TB had decreased testosterone levels in sera with modest increases of estradiol concentrations, may be viewed as an unsuccessful attempt to improve cell mediated immune protective mechanisms [45]. In contrast to these results in humans, our results showed that BALB/c tuberculous M mice increased testosterone serum levels in coincidence with progressive inflammation, from day 14 when granulomas start their formation until day 60, when substantial lung area is affected by pneumonia. These results suggest that testosterone might be involved in the modulation of inflammation, decreasing tissue damage by excessive inflammation. However, this modulation of inflammation might decrease the protective pro-inflammatory cytokines production favouring bacilli growth and disease progression. This statement was supported by our results from male mice castrated at late active disease (day 60), that showed lower pulmonary bacilli loads and higher expression of TNFα, IL-12 and IFN-γ than non-castrated mice.

In conclusion, M mice are more susceptible to TB than F mice. This higher susceptibility was prevented by castration before infection or during late disease, suggesting that testosterone is a potential susceptibility factor. These experimental results show that the endocrine systems, in this case the sexual hormones, substantially modifies the activity of the immune sytem and the inflammatory response influencing the course of experimental pulmonary TB.
